# Temporal patterns of cotton Fusarium and Verticillium wilt in Jiangsu coastal areas of China

**DOI:** 10.1038/s41598-017-12985-1

**Published:** 2017-10-03

**Authors:** Xiaogang Li, Ya’nan Zhang, Changfeng Ding, Wenhua Xu, Xingxiang Wang

**Affiliations:** 1 Key Laboratory of Soil Environment and Pollution Remediation, Institute of Soil Science, Chinese Academy of Sciences, Nanjing, 210008 China; 2 Jiangsu Coastal Region Institute of Agricultural Science, Yancheng, 224002 China

## Abstract

Cotton diseases caused by soil-borne pathogenic fungi present a major constraint to cotton production not only in China but also worldwide. A long-term field inventory was made of the prevalence of Fusarium and Verticillium wilt of cotton in the Jiangsu coastal area of China from 2000 to 2014. Various factors (crop varieties, rotation and weather) were analyzed to explore the dynamics of these diseases in cotton. The results showed that the prevalence of Fusarium and Verticillium wilt increased before 2005 and that Verticillium wilt remained at a high incidence over most of the past 10 years, while Fusarium wilt began to gradually decrease after 2005. The dynamics of Fusarium and Verticillium wilt were closely associated with the introduced cotton varieties and the intensive cropping history. In addition, weather conditions occurring during some of the years appeared to coincide with a substantial variation in the wilt diseases. Our study highlighted epidemiological dynamics of Fusarium and Verticillium wilt in a long-term survey.

## Introduction

Cotton (*Gossypium hirsutum* L.) is a globally important crop. China is the top producer of cotton in the world, and includes three planting regions: the Yellow River basin, the Yangtze River basin, and the Northwest Inland basin^1^. Nevertheless, Fusarium and Verticillium wilt of cotton caused by soil-borne pathogens frequently cause severe economic losses not only in China but also worldwide^2,3^. Both diseases were introduced into China in the 1930s, and spread through the main cotton planting regions in the 1970s. These pathogens began to cause severe disease problems in the early 1980s, causing the loss of more than 150 thousand tons of lint cotton per year in China^4^. Currently, Fusarium and Verticillium wilt are still the main obstacles for sustaining high yields and quality as well as stable production of cotton in China^5,6^.

*Fusarium oxysporum* f. sp. *vasinfectum* W.C. Synder & H.N. Hans (FOV), is the cause of cotton Fusarium wilt. FOV is composed of eight nominal races worldwide, and three of these races (3, 7, and 8) exist in China^4^. There are two races of *Verticillium dahliae* that infect cotton, which was classified as defoliating or nondefoliating based on symptoms^7^. These pathogens can exist in the forms of mycelia, chlamydospores, microsclerotia in soil and crop debris, and can persist in soil for extended periods until a new cycle of infection begins^4^. Both can also survive saprophytically on other crops and weeds. Fusarium wilt frequently initiates disease symptoms after the seedling stage and peaks at the squaring stage. The disease symptoms characteristically appear in patches between the main veins, with the rest of the leaf remaining green, resulting in chlorotic patches that gradually become necrotic and then leaf detachment (defoliation) from the stem^4^. Verticillium wilt occurs before the squaring stage and reaches a peak at the boll-setting stage with yellow mottled or defoliating symptoms^7^. However, the determinants that lead to the occurrence of these diseases are not yet fully understood^8,9^.

Available control measures are not completely effective, because diverse factors (agronomical, edaphic, climatic, pathogen population structure, cultivar susceptibility, etc.) contribute to the prevalence of soil-borne diseases (especially with management of Verticillium wilt)^10^. For instance, a broad range of hosts can be colonized by *V. dahliae*, thus prolonging the pathogen’s survival capacity in cotton soil^11^. Therefore, effective management of cotton Verticillium and Fusarium wilts requires an integrated management strategy. However, the complexity of an integrated approach requires a great deal of information about individual tactics as well as their interactions, and gaps in our understanding still exist^11,12^. Epidemiology is concerned with the patterns of disease occurrence and the factors that influence these patterns^13,14^. Thus, it is important to understand the specific epidemiologic characteristics of Fusarium and Verticillium wilt in regional areas for disease management. These characteristics can provide important clues to the ecology of diseases.

There are a number of key factors contributing to the distribution and expansion of cotton Verticillium wilt and Fusarium wilt in fields, and these factors can depend on the pathogen, the host and/or the environment. For example, Fusarium wilt severity was found to be closely related to the disease resistance of cotton and to soil temperature^15^. Early studies focused on the connections between certain cotton diseases and the potential individual causal factors over limited growing seasons of observation in the field. However, many of these relationships are still not well-understood, and thus long-term studies over successive years are needed. Moreover, these factors can interact in overlapping or synergistic ways^16–18^. Therefore, a broad and systematic survey of the relationships between cotton wilts, environmental factors, and important production factors such as cultivar susceptibility, crop rotations, etc. will be important in understanding the expansion of cotton diseases in fields^19,20^.

The objectives of the current study were to (i) investigate the prevalence and incidence of cotton Fusarium and Verticillium wilt over a 15-year consecutive time period and to (ii) analyze the characteristics of the disease occurrences, with the aim of providing a scientific basis for the development and evaluation of preventive crop protection measures.

## Results

### Cotton planting and disease prevalence

**Figure 1:**
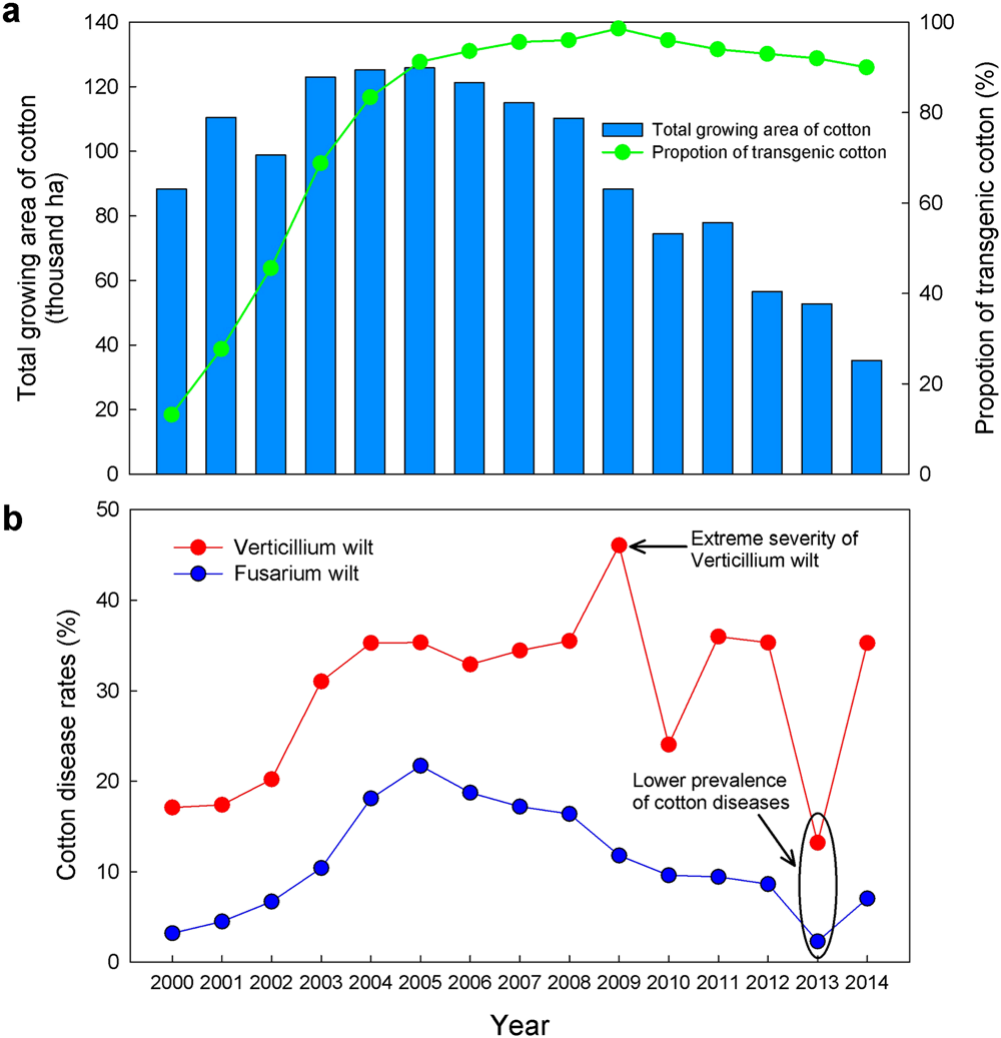
Size of cotton planting areas, proportion of transgenic cotton and disease prevalence of Verticillium wilt and Fusarium wilt. The size of the area indicate fields planted with cotton varieties in the Tinghu District, Dafeng county, Sheyang County, and Dongtai county (**a**). Proportion of transgenic cotton is a measure of the percentage of growing areas of transgenic cotton relative to the total areas of cotton in the surveyed regions (**a**). Disease prevalence is a measure of the proportion (or percentage) of geographical sampling units (cotton fields) in which the corresponding disease symptoms were observed relative to the total number of geographical sampling units (cotton fields) that were inspected (**b**). Arrows indicate extraordinary prevalence of cotton diseases.

**Figure 2:**
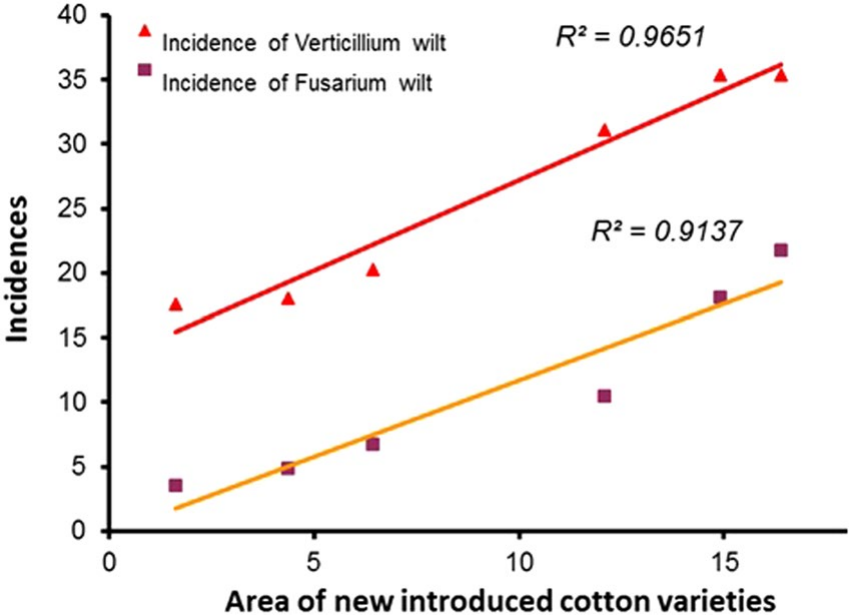
Correlation between areas of newly introduced cotton varieties and the incidences of Verticillium wilt and Fusarium wilt in the surveyed regions from 2000 to 2005. Area indicated the additional cotton varieties planted in the Tinghu District, Dafeng county, Sheyang County, and Dongtai county after 2000.

The size of the areas planted with cotton gradually increased from 2000 until 2005 and then showed a declining trend (Fig. 1a). The prevalence of Fusarium and Verticillium wilt increased from 2000 to 2005. From 2005 to 2014, the disease prevalence of Verticillium wilt behaved differently than Fusarium wilt. Verticillium wilt maintained a higher severity of disease prevalence than Fusarium wilt (>33%, except for the 2010 and 2013 growing seasons), and there was a wide year-to-year variation in incidence (Fig. 1b). In contrast, the prevalence of Fusarium wilt showed a gradual decrease from 2005 (Fig. 2b).

### Cotton varieties during 2000 to 2005 in cotton planting areas

Before the mid-1990s, cotton varieties were generally developed from the local major cultivars. Thereafter, many new cotton varieties were introduced into the main cotton planting areas due to the commercial use of transgenic insect-resistant cotton (Fig. 1a). The number of cotton varieties grown in a certain region was increased from 3 in 2000 to 9 in 2005 (Table 1). Our survey found that there were 33 different varieties that were grown on <67 ha; but only 13 varieties were grown on up to 333 ha in the region. Correlation analysis determined that cotton diseases had a significant positive relationship with the increasing planting areas of newly introduced varieties (Fig. 2).

**Table 1:** Main cotton varieties planted from 2000 to 2005 in the surveyed regions.

Plant years	Cotton varieties
2000	Sumian-9, Sumian-12, Sumian-15
2001	Sumian-9, Sumian-12, Sumian-15, Guokang-22
2002	Sumian-9, Sumian-12, Sumian-15, Zhongmian-29
2003	Sumian-9, Sumian-15, Sumian-22, Kemian-1, Luyanmian-15, Luyanmian-23, Zhongmian-29
2004	Sumian-9, Sumian-15, Sumian-22, Kemian-1, Keman-4, Luyanmian-15, Nankang-3, Zhongmian-29
2005	Sumian-9, Sumian-22, Kemian-1, Keman-3, Luyanmian-15, Luyanmian-20, Zhongmian-29, Fengkang-103, Nanmian-6

Note: Cotton varieties marked with gray level indicate the extra varieties increases compared to the previous season.

### Evaluation of disease incidences of various cotton varieties in field

The resistances of cotton varieties significantly differed for both Fusarium and Verticillium wilt (Table 2). Cotton varieties showed low incidences of Fusarium wilt with disease indices lower than 5. The incidence of Verticillium wilt on all tested cotton varieties significantly increased with a prolonged growth period (from July to September), and most tested varieties had a disease index of more than 35 in the later period of cotton growth (Table 2). Moreover, field survey in 220 fields (13 varieties) for both diseases indicated that Fusarium wilt incidence was <6% for all 13 varieties, while Verticillium wilt incidence was >20% for all but 5 varieties (Table S1).

**Table 2:** Assessment of the incidence and disease indices of cotton varieties in this field study.

Cotton Varieties	Fusarium wilt		Verticillium wilt					
	15 June		28 July		25 August		15 September	
	Incidence/%	Disease index	Incidence/%	Disease index	Incidence/%	Disease index	Incidence/%	Disease index
99B	1.28 ± 0.08b*	1.28 ± 0.08b	19.8 ± 1.5f	6.48 ± 0.7e	43.2 ± 3.1i	11.4 ± 0.09i	76.63 ± 6.3e	50.03 ± 4.7de
Zhong-29	1.25 ± 0.11b	1.25 ± 0.11b	5.3 ± 0.3d	1.84 ± 0.2d	33.7 ± 3.3h	9.47 ± 0.12h	83.05 ± 6.9f	74.23 ± 6.9f
Zhong-41	0a	0a	1.1 ± 0.1b	0.27 ± 0.03b	6.7 ± 0.6d	1.67 ± 0.15d	65.47 ± 5.9cd	48.47 ± 3.7e
Zhong-45	3.49 ± 0.13d	3.49 ± 0.13d	3.2 ± 0.2c	0.8 ± 0.04c	9.6 ± 0.6e	2.66 ± 0.23e	68.31 ± 6.3d	50.0 ± 3.6de
Lu-15	1.28 ± 0.07b	1.28 ± 0.07b	4.3 ± 0.2d	1.9 ± 0.08d	1.1 ± 0.05b	0.27 ± 0.03b	40.0 ± 3.51b	30.6 ± 2.9c
Lu-21	2.47 ± 0.15c	2.47 ± 0.15c	1.2 ± 0.05b	0.29 ± 0.03b	0a	0a	34.31 ± 2.4a	21.11 ± 1.6a
SGK321	1.14 ± 0.04b	1.14 ± 0.04b	4.2 ± 0.3d	1.84 ± 0.09d	13.7 ± 1.7f	4.47 ± 0.51f	40.94 ± 3.6b	33.51 ± 2.5c
Ji-668	2.5 ± 0.23c	2.5 ± 0.23c	3.2 ± 0.4c	1.06 ± 0.05d	13.8 ± 1.3f	3.46 ± 0.3f	41.47 ± 5.7b	29.4 ± 3.1bc
Xin-4	0a	0a	0a	0a	7.8 ± 0.9d	2.2 ± 0.21d	59.89 ± 3.9c	46.95 ± 3.6d
Han-109	1.22 ± 0.05b	1.22 ± 0.05b	3.1 ± 0.2c	1.8 ± 0.08d	3.1 ± 0.1c	0.77 ± 0.05c	42.31 ± 4.8b	28.75 ± 3.4bc
Yu-35	0a	0a	0a	0a	1.1 ± 0.09b	0.28 ± 0.01b	38.31 ± 5.1b	25.42 ± 2.6b
Xiang-3	0a	0a	18.6 ± 1.8f	8.43 ± 0.7f	34.9 ± 2.9h	11.3 ± 0.09i	63.68 ± 4.2c	57.52 ± 4.8e
Nan-3	3.49 ± 0.13d	3.49 ± 0.13d	9.5 ± 1.1e	6.05 ± 0.8e	26.3 ± 1.3g	10.8 ± 0.12hi	84.21 ± 6.9f	79.78 ± 6.5f
Kejian-30	0a	0a	5.4 ± 1.3d	1.88 ± 0.7d	8.6 ± 2.1d	2.69 ± 0.62d	46.9 ± 5.9b	37.29 ± 3.5c

Asterisks *indicates that the different letters in the same column showed significant difference among the surveyed varieties at *p* < 0.05.

### Cropping systems

The paddy-upland system of rice and cotton rotation accounted for a low percentage (0–5%) of the total cotton areas. However, the barley-cotton system accounted for the majority of the cotton area (Table 3). Most fields in the surveyed regions had been intensively cropped with cotton for many years. Between 15% and 20% of the fields had been intensively cropped with cotton in a rotation for more than 10 years (Table 3). Correlation analysis also determined that cotton diseases had a significant positive relationship with the cropping ages of cotton (Table 3).

**Table 3:** The association of planted acres with different cropping lengths and their effects on incidence of cotton diseases in 2012.

Cropping systems*	Ratio of different planting lengths				Effect of cropping ages of cotton^#^			
	0–2 y	3–5 y	6–10 y	>10 y	Fusarium wilt		Verticillium wilt	
					*r*	*p*	*r*	*p*
Barley-cotton	0–5%	15–25%	25–40%	15–20%	0.940	0.015	0.948	0.013

*Cropping systems exclude the paddy-upland rotation system, because it had low percentage (0–5%) of the total cotton areas compared to dry land system (Barley-cotton) in the surveyed region. # *r* indicates the coefficient of correlation, and *p* indicates the significance.

### Influence of extreme climatic condition on disease prevalence

**Figure 3:**
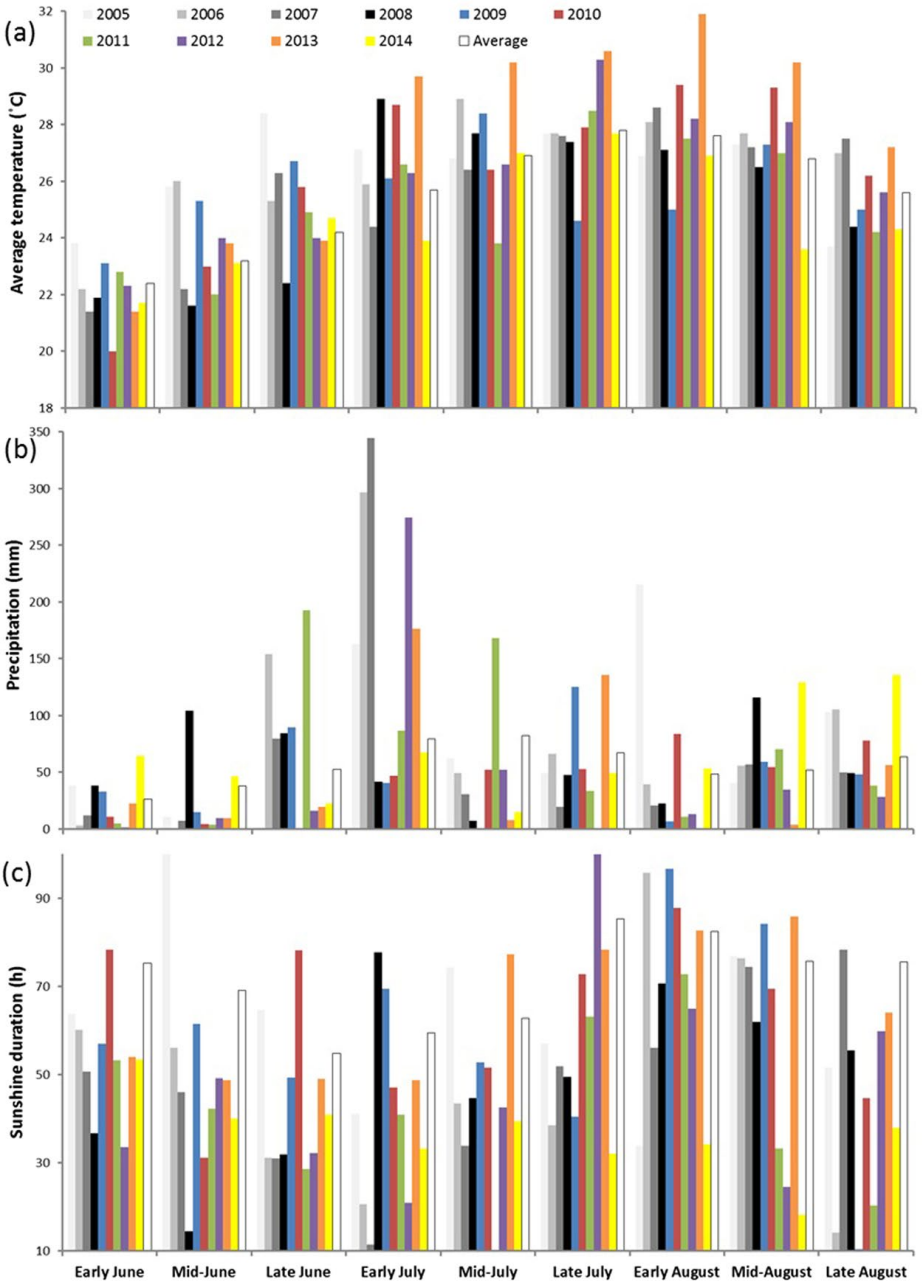
The climatic information for the surveyed regions from June to August of 2005 to 2014. (**a**): average temperature; (**b**): precipitation; (**c**): sunshine duration. “Average” in figure indicates the mean of the last 50 years. Meteorological dates came from Yancheng weather bureau.

During the past ten years, the prevalence of cotton diseases was relatively more serious in 2009, especially for Verticillium wilt (Fig. 1b). The continuous rainfall from late June to early August 2009 (Precipitation: >250 mm) provided suitable conditions for the growth and prevalence of cotton pathogens, causing initial outbreaks of cotton diseases (Fig. 3). The optimal temperature (≈25 °C), due to sunny weather after mid-August, resulted in further aggravation of cotton Verticillium wilt. In contrast, higher temperatures (≈30 °C) and less rainfall in July 2013 (Precipitation: <155 mm) than in ordinary years also contributed to the low disease prevalence (Fig. 3).

## Discussion

Our long-term continuous investigation of the cotton fields across the Jiangsu Coastal area showed that the prevalence of Fusarium and Verticillium wilt increased from 2000 to 2005, indicating a rapid spread of the diseases in the cotton fields. From 2005 to 2014, the development of the two cotton diseases followed different trends. Verticillium wilt maintained a high disease incidence, whereas the incidence of Fusarium wilt reduced gradually. The area of fields planted with cotton showed a declining trend from 2005, which confirmed the phenomenon that cotton Verticillium wilt was a significant constraint on cotton yields^21^.

Previous work on the region concluded that there were few observations of Fusarium wilt resulting in cotton death, and the incidence of Verticillium wilt was generally less than 10% during the period of 1995–2000^22,23^, which indicated that both cotton diseases had a lower prevalence before 2000. However, due to the commercial use of transgenic insect-resistant cotton, many new varieties have been introduced to the main cotton planting areas. These introduced cotton seeds may bring new strains of pathogens to the local region in cases of a lack of quarantine^23^. Significant positive correlations between the severity of cotton diseases and planting sizes of new cotton varieties further suggested that the introduction of more varieties could be one of the main reasons for the quick spread of cotton Fusarium wilt and Verticillium wilt from 2000 to 2005.

Severity of cotton Fusarium wilt and Verticillium wilt is closely associated with the disease resistance of cotton varieties^24,25^. Our field assessment recorded low incidences of Fusarium wilt for various cotton varieties. This result was in agreement with other studies^21^. A review of the disease resistance of cotton varieties from 1998–2011 concluded that most cotton varieties applied after 2008 had better resistance to Fusarium wilt but were susceptible to Verticillium wilt^26^. In this study, the cotton varieties tested had higher susceptibility to Verticillium wilt. These results comprehensively explained that the disease prevalence of Verticillium wilt behaved differently than Fusarium wilt from 2005 to 2014. This suggests no improvement in the resistance of cotton varieties due to the lack of cotton variety sources and the high variation of *V. dahliae* ^27,28^, either of which could be the cause of the prevalence of cotton Verticillium wilt in this region.

Rotation and fallow are rarely practiced in intensive agricultural production. Our field investigation on the cultivation history indicated that most cotton fields in the surveyed regions had been intensively planted with cotton for many years. Since FOV has a narrow host range, a barley-cotton rotation may slow the buildup of FOV^19^. Cotton Fusarium wilt is often associated with root-knot nematode (*Meloidogyne incognita*). Nevertheless, outbreaks of cotton nematode diseases have not been recorded in the surveyed region over the last years. In addition, *M. incognita* was seldom found in the cotton-growing region^29,30^, although it is of concern in the United States, India, Egypt and Brazil^31^. However, *V. dahliae* is able to infect a broad range of crop species, and there is existing cross-pathogenicity between cotton-infecting isolates and those from other hosts^10,32,33^. Intensive cropping of cotton led to the accumulation of pathogens in the soil, and changes to the soil microbial community composition favored the disease outcome^34,35^. Studies found that populations of *V. dahliae* and *F. oxysporum* in soils significantly increased with prolonged cropping of cotton and that the disease incidences were positively related to the number of pathogens in cotton cropping soils^36,37^. Therefore, intensive cropping is probably a major cause of disease prevalence, especially for cotton Verticillium wilt.

Climatic conditions can influence disease outbreaks in cotton fields^38^. Verticillium wilt occurs readily with air temperatures from 22–28 °C, slowly develops below 20 °C or above 30 °C, and symptoms don’t develop at temperatures over 35 °C^5,39^. Rain can aggravate the outbreak and prevalence by spreading and germinating spores of *V. dahliae* ^18,40^. *F. oxysporum* can successfully infect cotton root within 12 d when soil temperature is up to 28 °C, but it takes 24 d if soil temperature is below 25 °C^40^. The breakout of cotton Fusarium wilt also depends on soil moisture. The conversely climatic conditions of 2009 and 2013 partially explained the dynamics of cotton Verticillium wilt and Fusarium wilt. Therefore, including data on climatic conditions from June to August could be helpful in explaining the prevalence of both cotton diseases.

## Conclusions

This study provided data from a long-term inventory of the temporal patterns of Fusarium wilt and Verticillium wilt in cotton. The prevalence of Fusarium and Verticillium wilt had a substantial increase between 2000 and 2005, and cotton Verticillium wilt remained at a high prevalence through 2014. Investigation on the characteristics of the disease occurrences suggests that the introduction of cotton varieties with improved disease resistance combined with proper crop rotation practices should be applied to control soil-borne fungal diseases in cotton.

## Materials and Methods

### Selection of the surveyed areas

The Jiangsu Coastal area has historically had a high quality of cotton production within the Yangtze River cotton basin in China but also frequently suffered from diseases such as cotton wilt caused by Fusarium and Verticillium, which have recently become a major bottleneck in the development of local cotton production. This area (32°34′−34°28′N, 119°27′−120°54′E) is located in the middle of the east coast of China across the warm temperate zone and the northern subtropics. The annual average temperature is approximately 14 °C; the frost-free period is on average 220 days; and the annual precipitation amounts average 1,000–1,080 mm, with rainfall generally concentrated between June and August. Thus, the climatic conditions are suitable for planting cotton. The cultivation process widely adopted by the local farmers was that cotton seedlings were first raised in a nutrition bowl in the nursery and were then transplanted to the fields in May. The investigations were conducted in the Tinghu District, Dafeng county, Sheyang county, and Dongtai county in Jiangsu Province (eastern China). The four surveyed sites accounted for more than 70% of the Jiangsu coastal area production.

### Field surveys

**Figure 4:**
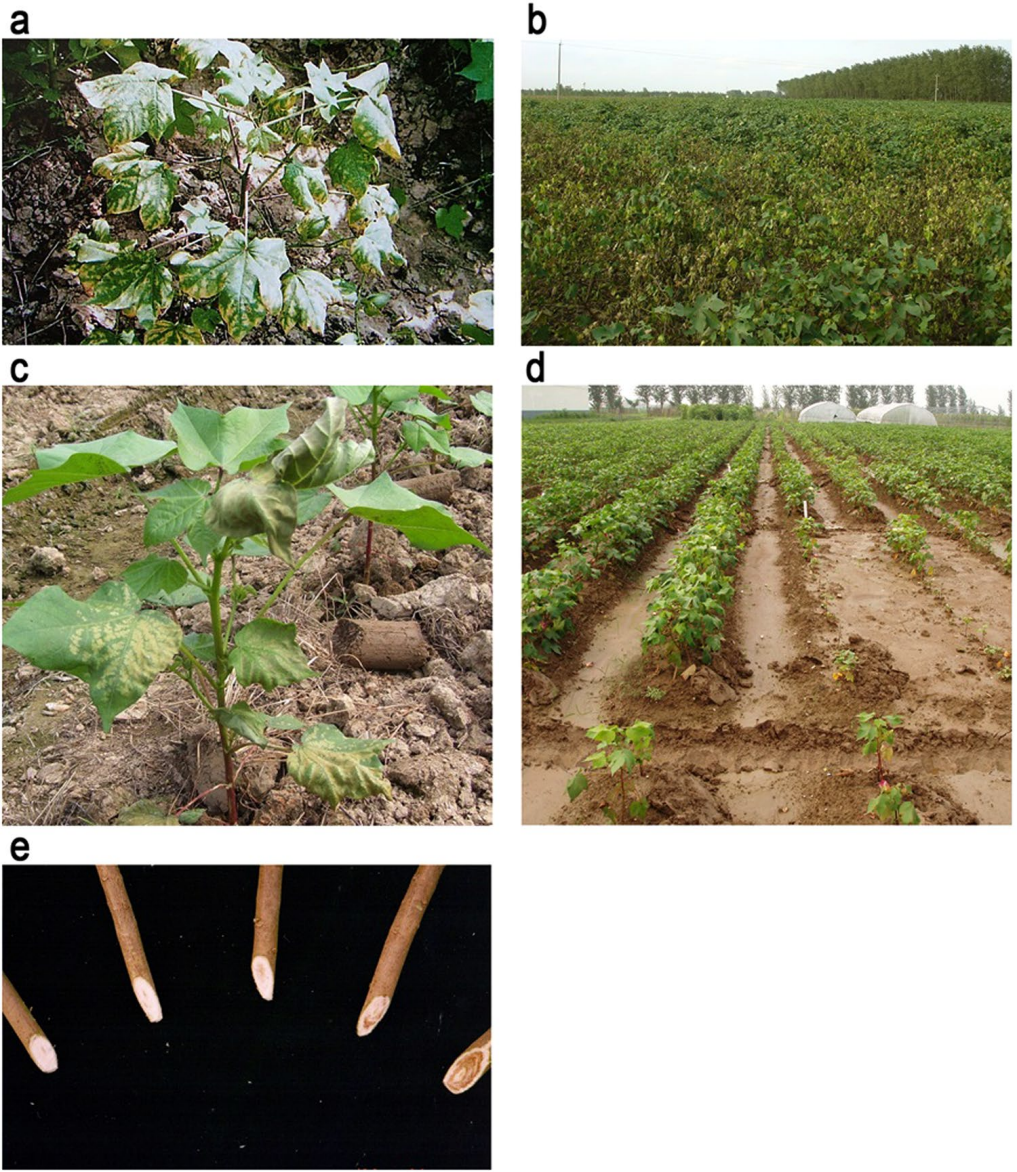
Cotton wilt caused by *Fusarium oxysporum* f. sp. *vasinfectum* and *Verticillium dahliae* Kleb. Verticillium wilt occurs before the squaring stage and reaches a peak at the boll-setting stage (**a**) with yellow mottled, defoliating symptoms (**b**). Fusarium wilt frequently occurs after the seedling stage and peaks at the squaring stage (**c**), during which symptoms include yellow, amaranthine, and green wilting types (**d**). The selected cotton plants were cut off to inspect wilt symptoms in lab (**e**).

Fusarium cotton wilt frequently occurs after the expansion of the first true leaf in mid to late June; thus, investigation of Fusarium wilt was conducted one month after sowing (May). Screening for symptoms of Verticillium wilt was started in mid-June and comprehensively recorded during the flowering and boll-setting stage. The surveys were conducted from 2000 to 2014, with a minimum of 100 fields investigated in each county at each surveying event. The surveyed cotton fields were owned by different farmers and were selected across the county. The sampling survey was designed in collaboration with the agricultural extension service of the Local Government, which supplied the information about the cotton fields including area (ha) and type of cultivars (Fig. 4).

To assess the intensity of the diseases, we measured disease prevalence^41^. Disease prevalence is a measure of the proportion (or percentage) of geographical sampling units (cotton fields) in which the corresponding disease symptoms were observed relative to the total number of geographical sampling units (cotton fields) that were inspected. Furthermore, we randomly collected cotton plants with representative symptoms in fields to identify the disease causal agent in the laboratory. Briefly, small internal fragments of petioles from different affected cotton plantings were surface-sterilized with HgCl_2_ (0.1%) for 5–8 min and subsequently rinsed with sterile water for 1 min. Segments were placed on a petri plate in water agar with chlortetracycline (30 mg L^−1^), and fungal colonies were identified based on the morphological characteristics.

### Evaluation of disease incidences of various cotton varieties in field

Cotton varieties that had been widely grown in the Jiangsu Coastal area were selected to assess their disease resistances. Cotton varieties included DP99B, Zhong-29, Zhong-41, Zhong-45, Lu-15, Lu-21, SGK321, Ji-668, Xin-4, Han-109, Yu-35, Xiang-3, Nan-3, and Kejian-30. In 2012, a field experiment was conducted at Cotton Raw Seed Growing Farm of Dafeng County, which has had a high incidence of cotton soil-borne diseases (Fusarium wilt and Verticillium wilt) for the last 5 years. Field trials were arranged in a randomized complete block design with three replicates per cotton variety. The plot size for each replication was 5 m by 6 m (30 m^2^) with 4 rows per plot. The density of *F. oxysporum* and *V. dahliae* microsclerotia was estimated to be 600 cfu g^−1^ dry soil and 2 × 10 cfu g^−1^ dry soil, respectively, based on their selective cultivation mediums. The planting scheme, fertilization, and all other field management practices used in the study were the same as those used in the local cotton fields.

The incidence of Fusarium wilt was evaluated after 30 d of planting in fields (June15). The degree of Fusarium wilt was estimated as follows: for level 0, the plant is healthy, no leaves have disease-like symptoms, and growth is normal; for level I, 1 to 2 cotyledons wilt and turn yellow; for level II, the first true leaf wilts and leaf veins appear to have yellow reticulation; for level III, the plant is stunted or wilts; and for level IV, the plant dies. The incidence of cotton Verticillium wilt was evaluated beginning at 60 days after planting in fields, and the frequency of evaluation occurred at intervals of 7 d until October. Plants that have died due to Fusarium wilt were not assessed for Verticillium wilt. Grading standards of Verticillium wilt used in the field investigation were as follows: for level 0, the plant is healthy, no leaves have disease-like symptoms, and growth is normal; for level I, less than one quarter of the leaves are yellow and withered, and the stem vascular bundle turns hazel; for level II, one quarter to one half of the leaves appear to have the same symptoms described as “I level”; for level III: one half to three quarters of the leaves appear to have the above symptoms; and for level IV, most leaves wither and fall, and the cotton plant dies.

### Survey of agronomic management of cotton fields

From 2012 to 2013 a questionnaire was sent to randomly chosen farmers who provided details about agronomic practices (e.g., age of the plantation, crop management), origin of their cotton seeds, previous cropping history and symptomatology for each cotton field surveyed. To reduce biases potentially introduced from the farmers, all cotton fields with wilt symptoms as identified by the farmers were visited by the investigators to validate survey results.

### Statistical analyses

Mean values of cotton disease prevalence were compared for different years. The incidence and disease indices of cotton varieties evaluated in the study were calculated by the following formulas^5^: Incidence (%) = (Number of disease plants/Total number of surveyed plants) × 100; Disease index(DI) = [∑(Number of diseased plants × corresponding level)/(Total number of surveyed plants × 4)] × 100.

Classical ANOVA and post hoc comparisons were performed to test for significant differences in the disease index among the survey time points. The susceptible variety Zhong-45 was selected as the control to adjust the DI of the evaluated cotton. The cotton fields surveyed in 2012 were first classified based on cropping ages in rotation (0–2 y, 3–5 y, 6–10 y, >10 y) to calculate the means of disease incidences for planting age categories, and their correlations with the planting ages were determined using SPSS 13.0 (SPSS Inc., Chicago, IL, USA).
